# Histochemical Differential Diagnosis and Polarization Optical Analysis of Amyloid and Amyloidosis

**DOI:** 10.1100/tsw.2006.35

**Published:** 2006-01-27

**Authors:** M. Bély

**Affiliations:** Department of Pathology, Policlinic of the Hospitaller Brothers of St. John of God at Budapest, Frankel Leo Street 17-19, Budapest, Hungary

**Keywords:** amyloidosis, polarization optical analysis, differential diagnosis

## Abstract

Amyloidosis is characterized by extracellular deposition of protein fibrils of chemically heterogeneous composition. Early recognition and identification of amyloid deposits allows an early start of therapy, which may entail a better prognosis. Congo red staining according to Romhányi (1971) is a highly specific and sensitive method for early microscopic recognition of amyloidosis. The main and most important types of amyloidosis may be distinguished by classic histochemical methods of performate pretreatment according to Romhányi (1979), or by KMnO_4_ oxidation according to Wright (1977) followed by Congo red staining and viewed under polarized light. Differences in the speed of breakdown (disintegration) of amyloid deposits according to Bély and Apáthy allow a more precise distinction of various types of amyloid.

### PROLOGUE

#### In Memoriam Professor G Romhányi (September 15, 1905 – August 29, 1991)

"A Great Hungarian Personality…Enthusiastic Teacher and Mentor of Thousands", as outlined by Kinga Karlinger in her memorial lecture of this charismatic personality at the IXth International Symposium on Amyloidosis held July 15–21, 2001 in Budapest, Hungary.

György Romhányi was professor of pathology at the University of Pécs (1951–1976), corresponding member of the Hungarian Academy of Sciences (1975–), honorary member of several international professional societies, and had awards conferred on him by numerous medical associations. He established and determined the course of amyloidosis research in Hungary in the 20th century. Although Romhányi is known as a pioneer in amyloid research, his contributions to it are but a part of his life's work. He ingeniously utilized the polarization microscope and almost all his contributions are based on this method. "Die Methode ist alles" (The method is everything) was his favorite quotation of Ludwig, and the "method", polarization microscopy, enabled him to investigate the submicroscopic structure of elastic fibers, the cell nucleus, and also had him anticipating the double helix of DNA.

We pay homage to his memory with this paper.

Reference: Gógl, Á. and Karlinger, K. (2001) A great Hungarian personality: Professor Romhányi, enthusiastic teacher and mentor of thousands. *Amyloid: J. Protein Folding Disord.* **8(Suppl 2),** 177. (Guest Editor: Bély, M.)

### INTRODUCTION

Amyloidosis is characterized by extracellular deposition of protein fibrils of chemically heterogeneous composition. The classification of amyloid is based on the types of amyloid fibril proteins and their precursors[1,2]. Several diseases or disorders may be complicated by systemic or localized deposition of amyloid proteins (Table 1.).

These amyloid deposits are different, depending on (a) the type and (b) the stage of amyloidosis[3,4].

The aim of the present study was to demonstrate, by the classical histochemical methods of Romhányi[5] or Wright[6], the differentiation of systemic and localized amyloidosis using Congo red staining[7] after degradation of deposited amyloid according to Bély and Apáthy[8,9].

### MATERIAL AND METHODS

Fifty-seven systemic cases of systemic secondary AA amyloidosis were studied in a randomized autopsy population of 308 in-patients with various autoimmune diseases. Amyloid A protein deposits were present in 52 of 234 patients with rheumatoid arthritis (RA), in 2 of 50 with systemic lupus erythematosus, in 2 of 12 with psoriatic arthritis, and in 1 of 12 with progressive systemic sclerosis. The patients died at the National Institute of Rheumatology between 1970 and 1999. Systemic immunoglobulin light-chain (AL-λ, or κ) amyloidosis was studied in a selected autopsy population of 5 in-patients with B-cell lymphoma. Systemic senile amyloidosis with amyloid transthyretin deposits (ATTR) was found and analyzed in an 85-year-old autopsy patient, and systemic β2-microglobulin–related amyloidosis (Aβ2M), associated with hemodialysis was in a further one[10]. Isolated cerebral amyloid β protein deposits (Aβ) wer studied in a selected autopsy population of 12 in-patients with Alzheimer's disease, who died at the National Institute of Psychiatry and Neurology[11].

Regional, exclusively mediastinal amyloid A protein (AA) deposits were detected with bronchiolo-alveolar carcinoma in one of 234 RA patients. We found renal retention cysts containing concentrated β2-microglobulin glomerular filtrate (localized-Aβ2M) in 2 of 52 RA patients in combination with coexistent AA amyloidosis[4]. Islet amyloid polypeptide (AIAPP) localized to the islets of Langerhans were present in 16 of 101 pancreas, corpus amylaceum of the lung in 8 of 169, and corpus amylaceum of the prostate in 7 of 13 RA autopsy patients. Other forms of tissue (organ)–limited, localized amyloids were sporadically found in routine biopsy material.

The tissue specimens were fixed in 8% formaldehyde and embedded in paraffin. Serial sections were cut and stained with Hematoxylin and Eosin or Congo red according to Romhányi (1971), without alcoholic differentiation, and sealed with gum arabic.

The amyloid deposits were histochemically characterized by Congo red staining after performate pretreatment — 85% HCOOH (8 ml), 30% $H_2O_2$ (3 ml), and 96% $H_2SO_4$ (220 μl) according to Romhányi (1979) at 20°C for 1, 5, 10, 15, or 20 sec[7,8], or after oxidation (0.25% $KMnO_4$ and 0.15% $H_2SO_4$, 1/1)–induced degradation of amyloid deposits according to Wright et al. (1977) at 20°C for 30 sec, and 1, 3, 5, or 10 min[7,8]; covered with gum arabic and viewed by Olympus BX51 polarized light microscopy.

The classical histochemical results were confirmed by immunohistochemical staining using the streptavidin-biotin complex/horseradish peroxidase method (when specific antibodies were available). Endogenous peroxidase activity was inhibited by pretreatment with 3% $H_2O_2$-methanol for 20 min at 20°C, and nonspecific protein binding was inhibited by incubation in a solution of 5% human albumin for 20 min at 20°C. The sections were then incubated at 4°C for 12 h with the primary antibody, antihuman amyloid A-component dilution, 1:100 (monoclonal antibody MO759; DAKO, Glostrup, Denmark), antihuman amyloid P-component dilution, 1:200 (polyclonal antibody; DAKO, Glostrup, Denmark), antihuman β amyloid dilution, 1:300 (polyclonal antibody; DAKO, Glostrup, Denmark), a-hu β2-mikroglobulin 1:400 (polyclonal antibody A0072; DAKO, Glostrup, Denmark), antihuman λ-light chain

(polyclonal antibody N1569; DAKO, Glostrup, Denmark), or κ-light chain diluted (polyclonal antibody N1568; DAKO, Glostrup, Denmark), followed by incubation with a biotinylated secondary antibody (Multilink; BioGenex, San Ramon, CA) for 20 min at 20°C. Streptavidin-biotin complexes were visualized with the use of diaminobenzidine and $H_2O_2$ to detect peroxidase activity (15 min at 20°C).

**TABLE 1**
**The Main Types of Systemic and Localized Amyloidosis**
**(Precursor and Abbreviation of Amyloid Protein in Parenthesis)**

**1. Systemic (generalized) amyloidosis**
- 1.1. **Secondary (reactive)** - ((Apo)serumAA, SAA - serum amyloid A - **AA**)
- 1.2. **Primary** (myeloma-associated, B-cell dyscrasia) (immunoglobulin light chain - **AL**: λ-chain, κ-chain, immunoglobulin heavy chain - **AH**)
- 1.3. **Senile** - (transthyretin - **ATTR**)
- 1.4. **Hemodialysis-associated** - (β2-microglobulin - **Aβ2M**)
- 1.5. **Hereditary**
  - Familial Mediterranean fever (SAA - **AA**)
  - Familial amyloid polyneuropathy (transthyretin - **ATTR**)
  - Other hereditary forms of amyloidosis (apolipoprotein AI - **AApoAI**, apolipoprotein AII - **AapoAII,** apolipoprotein AIV - **AapoAIV,** gelsolin - **AGel,** lysozyme - **ALys**, cystatin C - **ACys**, fibrinogen alfa-chain - **AFib**)

**2. Organ(tissue)–limited (isolated, localized) amyloidosis**
- 2.1. **Cerebral**
  - β Protein-related amyloidosis (Alzheimer's disease) (Aβ protein precursor - **Aβ**)
    - Cerebral extravascular amyloidosis - cortical "amyloid plaque"
    - Sporadic cerebral amyloid angiopathy
  - Non-β protein-related amyloid diseases
    - Prion protein (PrP) amyloidosis (**APrP**): kuru, fatal familial insomnia, Creutzfeld-Jakob disease, Gerstmann-Sträussler-Schenker disease, PrP cerebral angiopathy
  - Aging pituitary (Prolactin - **APro**)
- 2.2. **Dystrophic (aging related)**
  - Localized to articular cartilage (?)
  - Localized to articular capsule, or ligaments (transthyretin - **ATTR**)
  - Localized to intervertebral discs
  - Localized to vertebral ligaments (transthyretin - **ATTR**)
- 2.3. **Endocrine related** - prohormone fragments
  - Localized to islets of Langerhans (Islet amyloid polypeptide - **AIAPP**)
  - Cardiac - atrial myocyte associated (atrial natriuretic prohormone-peptide - **AANF**)
  - Localized to parathyroid gland (parathormone prohormone?)
  - Localized to endocrine tumors
  - Medullary carcinoma of thyroid gland (C-cell thyroid tumors) ((Pro)calcitonin - **ACal**)
- 2.4. **Localized to epithelial tumors** (keratin)
  - Basal cell carcinoma
  - Calcifying epithelioma of Malherbe (Pilomatrixoma)
  - Squamous cell carcinoma
  - Calcifying epithelial odontogenic tumor of Pindborg
- 2.5. **Localized amyloidosis caused by concentrated secretion, or filtrate**
  - Seminal vesicle (concentrated secretion in seminal vesicles)
  - Prostate (concentrated secretion in prostatic glands)
  - Colloid cyst of thyroid gland (concentrated glandular secretion - ?)
- 2.6. **Localized amyloidosis caused by concentrated secretion, and/or of inflammatory origin**
  - Renal retention cysts (concentrated glomerular filtrate - β2-microglobulin - **Aβ2M**)
  - Prostatic corpora amylacea (β2-microglobulin - **Aβ2M**)
  - Pulmonary corpora amylacea (β2-microglobulin - **Aβ2M**)
- 2.7. **Solitary plasmocytoma** (Focal plasma cell, B-cell dyscrasia related, **AL**: λ-chain, κ-chain, **AH**)
- 2.8. **Cornea** (Lactoferin – **ALac**?)
- 2.9. **Iatrogenic** (Insulin - **AIns**)

### RESULTS

The histochemical characteristics of systemic and localized amyloidosis are summarized in Table 2.

- **The oriented array (arrangement, settlement) of amyloid filaments and fibrils in amyloid deposits induces a birefringence of typical apple green polarized color.**

There is *no pathognostic difference* in color of polarized light produced by biochemically heterogeneous amyloid deposits.

- **Different oxidative agents (performate, $KMnO_4$, etc.) may disintegrate the oriented arrangement of amyloid filaments and fibrils; consequently, the birefringence with typical apple green polarized color decreases, or disappears, depending on the applied oxidative agents, and depending on the time of destruction.**

For example, *systemic* or *localized amyloid A deposits* are very **sensitive to performate pretreatment**, birefringence disappears within 1 sec; on the other hand, these deposits are relatively **resistant to $KMnO_4$ oxidation**, destruction takes a longer time, birefringence disappears after 30 sec – 1 min only.

- **Performate is a more aggressive destructive agent than $KMnO_4$ oxidation.**

*Systemic amyloid A* deposits will disintegrate within 1 sec after **performate pretreatment**, whereas *AL-λ, or κ amyloid* takes more than 5 sec, *Aβ2M* more than 15 sec, *ATTR* more than 25 sec to disintegrate.

Disintegration of *systemic amyloid A* deposits takes at least 1 min, that of *systemic AL-λ, or κ amyloid* deposits at least 3 min, that of *Aβ2M* at least 30 sec, and disintegration of *ATTR* amyloid deposits takes at least 10 min of **$KMnO_4$ oxidation**.

After **performate pretreatment** *localized amyloid A* deposits will disintegrate within 1 sec, but *localized AL-λ, or κ amyloid* deposits are resistant to performate pretreatment for 20 sec (at least), *localized Aβ2M* 20 sec (at least), *dystrophic articular cartilage amyloid* for 3 sec.

Disintegration of *localized amyloid A* deposits takes at least 3 min, *localized AL-λ, or κ amyloid* deposits at least 5 min, and *dystrophic articular cartilage amyloid* deposits at least 15 min of **$KMnO_4$ oxidation**.

- **Amyloid deposits are more sensitive to performate pretreatment than to $KMnO_4$ oxidation.**

Any form of *amyloid A* deposits *(systemic* or *localized)* is very sensitive to **performate pretreatment**; *amyloid A* deposits disintegrate within 1 sec.

Any form of *amyloid A* deposits *(systemic* or *localized)* are resistant to **$KMnO_4$ oxidation** for 30 sec – 1 min.

The *systemic AL-λ, or κ amyloid amyloid* deposits are resistant to **performate pretreatment** for 1–5 sec, and the *localized AL-λ, or κ amyloid* deposits for 1–20 sec.

The *systemic AL-λ, or κ amyloid amyloid* deposits are resistant to **$KMnO_4$ oxidation** for 1–3 min, and the *localized AL-λ, or κ amyloid* deposits for 1–10 min.

The *systemic Aβ2M* deposits are resistant to **performate pretreatment** for 1–15 sec, and the *localized Aβ2M* deposits for 1–20 sec.

Any form of *Aβ2M* deposits *(systemic* or *localized)* are very sensitive to **$KMnO_4$ oxidation**: *systemic,* or *localized Aβ2M* deposits disintegrate within 30 sec.

- **Deposits in systemic amyloidosis are more sensitive to performate pretreatment or to $KMnO_4$ oxidation than localized forms of deposits.**

*Systemic AL-λ, or κ amyloid* deposits are resistant to **performate pretreatment** for 10 sec (or more), while the *localized AL-λ, or κ amyloid* deposits are resistant to **performate pretreatment** for 20 sec (at least).

*Systemic Aβ2M* deposits are resistant to **performate pretreatment** for 15 sec (or more), while the *localized Aβ2M* deposits are resistant to **performate pretreatment** for 20 sec (at least).

*Systemic AL-λ, or κ amyloid* deposits are resistant to **$KMnO_4$ oxidation** for 3 min, while the *localized AL-λ, or κ amyloid* deposits are resistant to *$KMnO_4$ oxidation* for 5 min (at least).

- **The minimal (early stage), "fresh" amyloid deposits are more sensitive to performate pretreatment or to $KMnO_4$ oxidation than massive (late stage), "old" amyloid deposits.**

Applying the same destructive agent, minimal amounts of amyloid (in case of any types of amyloid) disappear earlier than massive deposits

**TABLE 2**
**Histochemical Characteristics of Systemic and Localized Amyloidosis (According to the Time of Degradation by Performate, or $KMnO_4$)**

| | | | | | |
|---|---|---|---|---|---|
| **I. Performate pretreatment** | | | | | |
| **Systemic amyloidosis** | | | | | |
| **Type of amyloid protein/Time** | **1 sec** | **5 sec** | **10 sec** | **15 sec** | **20 sec** |
| **AA n = 57** | **S** | **S** | **S** | **S** | **S** |
| **AL-λ, or κ n = 5** | **R (1–5 sec)** | **R** | **R/S*** | **S*** | **S*** |
| **Aβ2M (β2-microglobulin) n = 1** | **R (1–15 sec)** | **R** | **R** | **R** | **R/S** |
| **ATTR (Senile) n = 1** | **R (1–25 sec)** | **R** | **R** | **R** | **R** |
| **Organ (tissue)-limited (localized, isolated) amyloidosis** | | | | | |
| **Type of amyloid protein/Time** | **1 sec** | **5 sec** | **10 sec** | **15 sec** | **20 sec** |
| **AA (Regional) n = 1** | **S** | **S** | **S** | **S** | **S** |
| **AL-λ, or κ n = 5** | **R (1–20 sec)** | **R** | **R** | **R** | **R** |
| **Aβ2M** (Renal retention cyst) **n = 2** | **R (1–20 sec)** | **R** | **R** | **R** | **R** |
| **Dystrophic** | | | | | |
| (Articular cartilage) **n = 25** | R (1–3 sec) | R/S | R/S | S | S |
| (Joint capsule) **n = 25** | R (1–10 sec) | R | R | R/S | R/S |
| **Aβ (Alzheimer's disease) n = 12** | **R (1–20 sec)** | **R** | **R** | **R** | **R** |
| **AIAPP** (Islet of Langerhans) **n = 16** | **R (1–10 sec)** | **R** | **R** | **R/S** | **R/S** |
| **Corpus amylaceum** (Lung) **n = 8** | **R (1–20 sec)** | **R** | **R** | **R** | **R** |
| **Corpus amylaceum** (Prostate) **n = 7** | **R (1–5 sec)** | **R** | **R/S** | **R/S** | **S** |
| **II. $KMnO_4$ oxidation** | | | | | |
| **Systemic amyloidosis** | | | | | |
| **Type of amyloid protein/Time** | **30 sec** | **1 min** | **3 min** | **5 min** | **10 min** |
| **AAa n = 60** | **R (30 sec – 1 min)** | **R***** | **R/S** | **R/S** | **S** |
| **AL-λ, or κ n = 5** | **R (1–3 min)** | **R** | **R** | **R/S** | **S** |
| **Aβ2M (β2- microglobulin) n = 1** | **R (30 sec)** | **R/S** | **S** | **S** | **S** |
| **ATTR (Senile) n = 1** | **R (1–10 min)** | **R** | **R** | **R** | **R** |
| **Organ (tissue)-limited (localized, isolated) amyloidosis** | | | | | |
| **Type of amyloid protein/Time** | **30 sec** | **1 min** | **3 min** | **5 min** | **10 min** |
| **AAa n = 1** | **R (30 sec – 1 min)** | **R***** | **R/S** | **R/S** | **S** |
| **AL-λ, or κ n = 5** | **R (30 sec – 10 min)** | **R** | **R** | **R**** | **R**** |
| **Aβ2M** (Renal retention cyst) **n = 2** | **S (30 sec)** | **S** | **S** | **S** | **S** |
| **Dystrophic** | | | | | |
| (Articular cartilage) **n = 25** | R (30 sec – 15 min) | R | R | R | R |
| (Joint capsule) **n = 25** | R (30 sec – 10 min) | R | R | R | R |
| **Aβ (Alzheimer's disease) n = 12** | **R (30 sec – 15 min)** | **R** | **R** | **R** | **R** |
| **AIAPP** (Islet of Langerhans) **n = 16** | **R (30 sec – 10 min)** | **R** | **R** | **R** | **R** |
| **Corpus amylaceum** (Lung) **n = 8** | **R (30 sec – 1 min)** | **R** | **R/S** | **S** | **S** |
| **Corpus amylaceum** (Prostate) **n = 7** | **R (30 sec – 10 min)** | **R** | **R/S** | **R** | **R** |

**Abbreviations:** R – resistant, S – sensitive.

* Advanced stage of systemic AL amyloidosis (at death) may be resistant to performate pretreatment (for 1–20 sec).

** Localized AL amyloid deposits recognized in early stage clinically may be R/S to $KMnO_4$ oxidation (for 5–10 min).

*** AA amyloid deposits may be R/S to $KMnO_4$ oxidation (for 1 min) in early stage of amyloidosis.

### *Remarks to Table 2, and Suggested Guideline for Differential Diagnosis of Amyloidosis*

- Five sections, serially cut, may be necessary for the histochemical differential diagnosis of the main types of amyloid.
- The histochemical diagnosis may be confirmed by immunohistochemical reaction on additional slides. Antihuman amyloid A reaction is specific, but minimal deposits of amyloid A may be missed with it. The minimal deposits of amyloid, viewed under polarized light, by their intensive green color in a dark field are also specific, but easier to see. The immunohistochemical analysis of AL, or β2-microglobulin reaction may be difficult especially in case of minimal deposits, because of the extensive background staining.
- Different types of amyloid may exist in the same patient, or side by side in the same organ, or tissue.

### *Suggested Schedule for Histochemical Analysis of Amyloid Deposits*

1. Step (First slide).

***Hematoxylin-Eosin staining***

2. Step (Second slide).

***Congo red staining according to Romhányi*** (1971), in order to confirm the suspected amyloidosis.

3. Step (Third slide).

**Performate pretreatment for 1 sec**

***Performate pretreatment for 1 sec*** is a very sensitive and specific method to recognize the AA amyloidosis. Any form of ***amyloid A protein deposit*** (systemic, or regional) is very ***sensitive*** to performate pretreatment. The birefringence of amyloid A deposits immediately disappears in slides stained with Congo red and viewed under polarized light; while other forms of amyloid deposits (***AL, ATTR, Aβ2M, Aβ***) are ***resistant*** and retain the specific green color under polarized light.

Performate pretreatment — applying different times for degradation — would be sufficient alone to identify the various types of systemic amyloidosis. $KMnO_4$ oxidation presents a second independent confirmation of the diagnosis, based on performate pretreatment.

- Systemic AA amyloidosis starts in the gastrointestinal tract[12]. AA amyloidosis is a systemic, progressive, cumulative process, and is present only in the gastrointestinal tract (“regionally”) in its early stage. The patient may die in this stage (because of other reasons, than amyloidosis), and the amyloidosis looks like “regional”.
   - Regional AA amyloidosis is very rare, and the diagnosis is difficult to prove. Special local reason (in our case malignancy) is necessary and a detailed autopsy is required.

4. Step (Fourth slide).

**$KMnO_4$ oxidation for 5 min**

***Systemic senile amyloidosis*** is ***resistant for 5 min*** $\boldsymbol{KMnO_4}$ ***oxidation*** (under polarized light the specific green color still exists).

***Systemic AL amyloidosis*** is ***resistant/sensitive for 5 min*** $\boldsymbol{KMnO_4}$ ***oxidation*** (under polarized light the specific green color decreases), while of β2-microglobulin deposits will be disintegrated (***Aβ2M*** is ***sensitive for 5 min*** $\boldsymbol{KMnO_4}$ ***oxidation*** (under polarized light the specific green color disappears).

$KMnO_4$ oxidation alone (without performate pretreatment) is not sufficient to differentiate between systemic AA and systemic AL amyloidosis, because the resistance (sensitivity) of AA and AL deposits may be practically the same.

- The systemic distribution of AL amyloid deposits should be confirmed by a second biopsy, for example, abdominal, subcutaneus fatty tissue biopsy (skin is often positive in systemic AL amyloidosis).
- Regional AL amyloid deposits are typically localized to the *respiratory tract* (e.g., pharynx, trachea, bronchi) or *urogenital tract* (e.g., urethra), and to the *skin* (e.g., amyloid tumor). The *localized AL deposits are more resistant to* $KMnO_4$ *oxidation (for 30 sec – 10 min), than the systemic immunoglobulin light (rarely heavy) chain deposits*. This is likely due to the fact that localized amyloid deposits cause of clinical symptoms only in later stages of the disease, and are diagnosed only in advanced stages of deposition. In advanced stages of amyloidosis the deposits are characterized by mature, electron microscopically closely packed, and fragmented filaments, which are more resistant to degradation[9,10].

5. Step (Fourth slide).

**KMnO4 oxidation for 10 min**

Systemic senile amyloidosis is resistant for 10 min $KMnO_4$ oxidation; while systemic AL amyloid deposits are sensitive (loss of specific green color with polarized light).

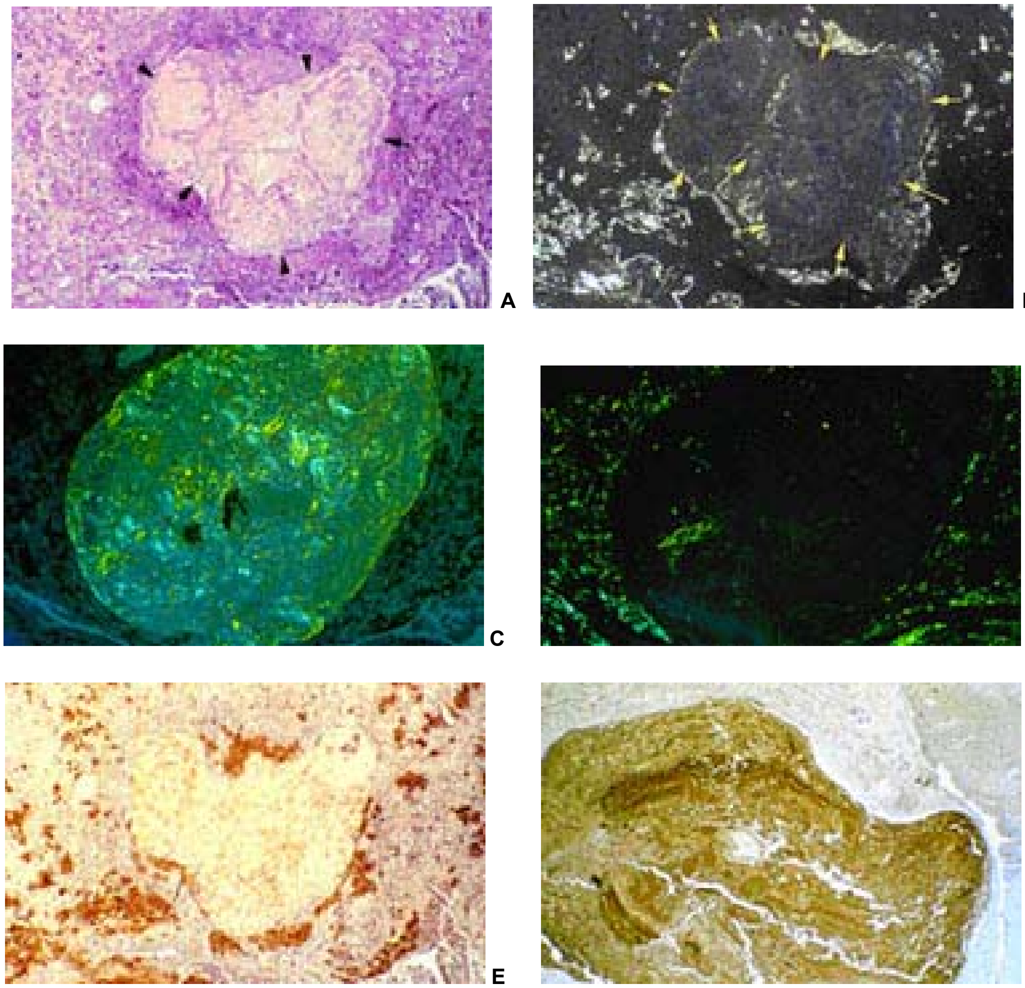

**FIGURE 1A–F.** Kidney. Systemic secondary AA amyloidosis and localized β2-microglobulin amyloid (Aβ2M) deposits. (A) Hematoxylin and Eosin. ×50. Aβ2M containing cyst is marked with arrows. This cyst corresponds to Fig. 1C (in which the β2-microglobulin amyloid deposits preserved the birefringence of typical green polarization color after performate pretreatment), and corresponds to Fig. 1F (in which the β2-microglobulin amyloid was stained with antihuman β2-microglobulin reaction). The rest of amyloid deposits in Fig. 1B are amyloid A (which have lost the birefringence of typical green polarization color after performate pretreatment in Fig. 1C; and preserved it after $KMnO_4$ oxidation in Fig. 1D; and was stained with antihuman amyloid A in Fig. 1E. (B) Same as Fig. 1A. Amyloid A and Aβ2M deposits with typical green polarization color. Congo red staining according to Romhányi, without alcoholic differentiation, covered with gum arabic, viewed under polarized light. ×50. (C) Same as Fig. 1A. **Amyloid A** deposits are **sensitive** to **performate pretreatment** (**for 1 sec)**, and **Aβ2M** deposits localized to renal retention cyst are **resistant** (**for 1–20 sec)**. ×50. (D) Same as Fig. 1A. **Amyloid A** is **resistant** to **$KMnO_4$ oxidation (for 30 sec – 1 min)**, and localized **Aβ2M** deposits are **sensitive** to $KMnO_4$ oxidation (**for 30 sec).** ×50. (E) Same as Fig. 1A. **Amyloid A deposits are positive for antihuman amyloid A component** 1:100 (monoclonal antibody MO759; DAKO, Glostrup, Denmark), Streptavidin-biotin complex/horseradish peroxidase reaction. ×50. (F) Localized **Aβ2M is positive for a-hu β2-microglobulin** 1:400 (polyclonal antibody A0072; DAKO, Glostrup, Denmark), Streptavidin-biotin complex/horseradish peroxidase reaction. ×50

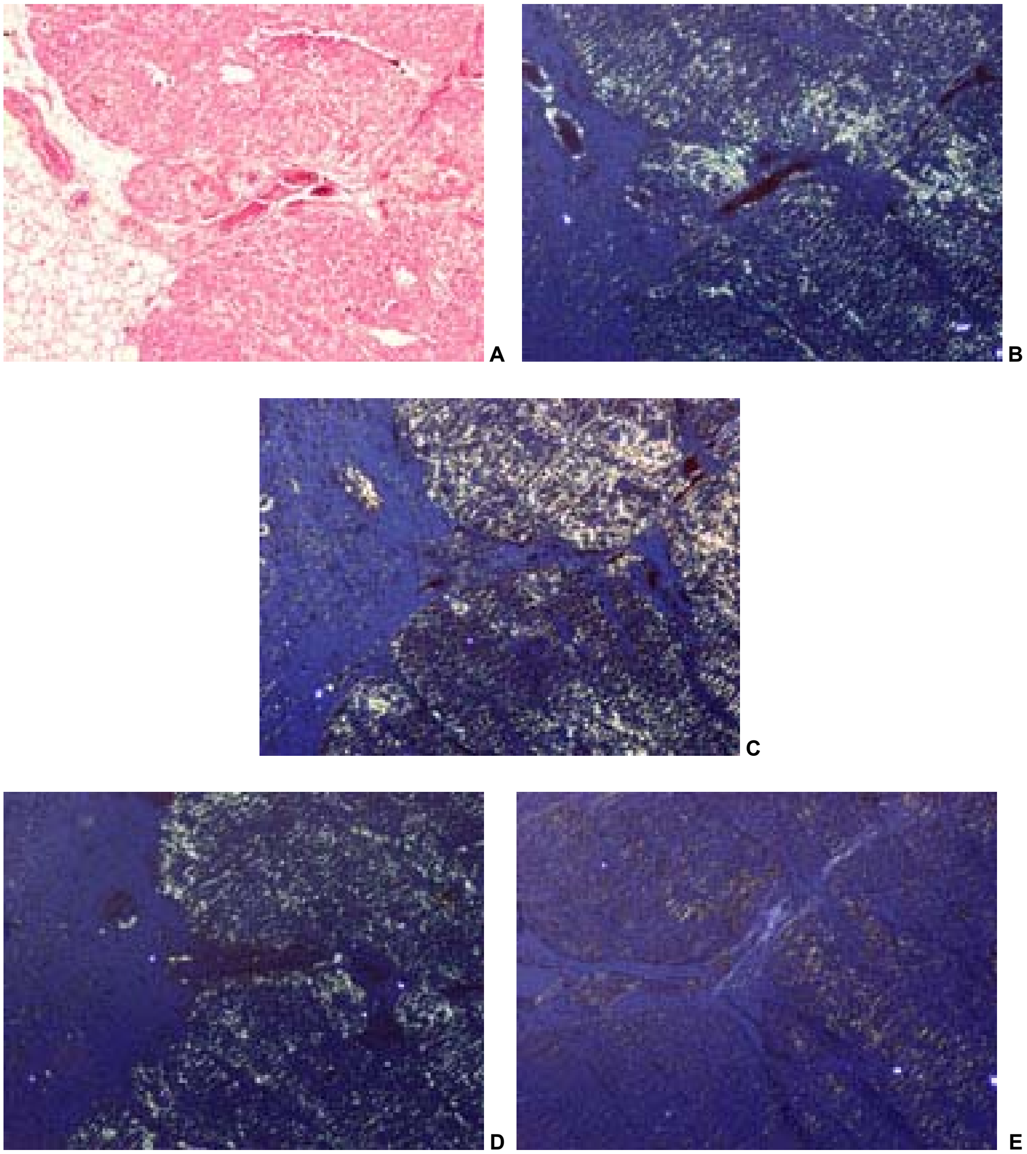

**FIGURE 2A–E.** Heart, subepicardial region. Systemic AL-κ amyloidosis. (A) Hematoxylin and Eosin. ×40. (B) Same as Fig. 2A. Congo red staining according to Romhányi, without alcoholic differentiation, covered with gum arabic, viewed under polarized light. ×40. (C) Same as Fig. 2A. Performate pretreatment (for 1 sec), Congo red staining, viewed under polarized light. **Systemic AL amyloid deposits** are **resistant** to **performate pretreatment** (**for 1–5 sec).** ×40. (D) Same as Fig. 2A. $KMnO_4$ oxidation **(for 1 min). Systemic AL amyloid deposits** are **resistant** to **$KMnO_4$ oxidation (for 30 sec – 3 min), resistant**/**sensitive** (**for 5 min),** and **sensitive (for 10 min).** ×40. (E) Same as Fig. 2A. $KMnO_4$ oxidation **(for 5 min). Systemic AL amyloid deposits** are **resistant**/**sensitive** to **$KMnO_4$ oxidation (for 5 min).** ×40.

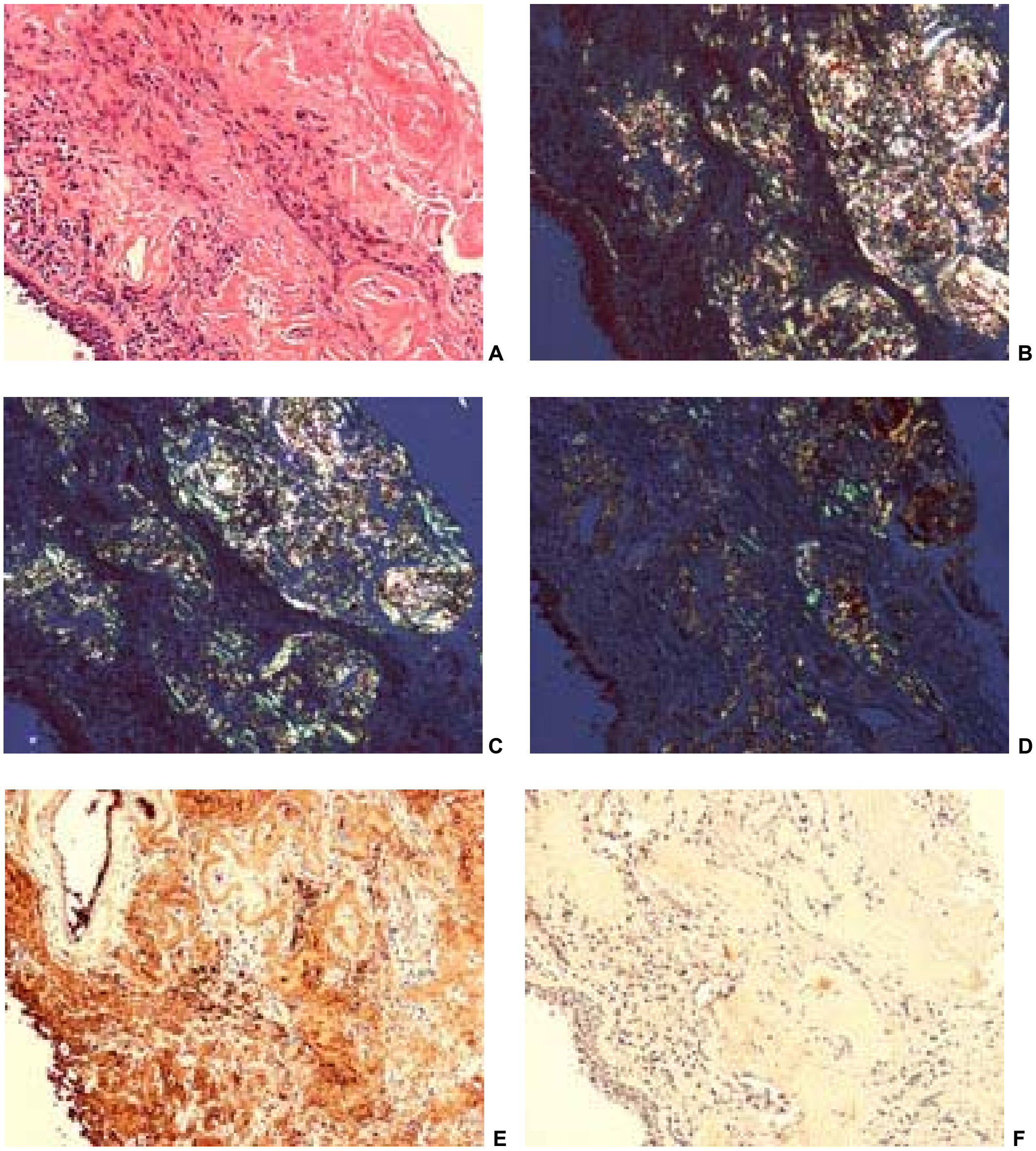

**FIGURE 3A–F.** Trachea. Localized AL-λ amyloidosis. (A) Hematoxylin and Eosin. ×100. (B) Same as Fig. 3A. Congo red staining according to Romhányi, without alcoholic differentiation, covered with gum arabic, viewed under polarized light. ×100. (C) Same as Fig. 3A. Performate pretreatment (for 1 sec), Congo red staining, viewed under polarized light. **Localized AL amyloid deposits** are **resistant** to **performate pretreatment** (**for 1–20 sec).** ×100. (D) Same as Fig. 3A. $KMnO_4$ oxidation **(for 5 min). Localized AL amyloid deposits** are **resistant** to **$KMnO_4$ oxidation (for 1 min – 10 min).** ×100. (E) Same as Fig. 3A. **Localized AL amyloid deposits** are **positive for** antihuman **λ-light chain** (polyclonal antibody N1569; DAKO, Glostrup, Denmark), Streptavidin-biotin complex/horseradish peroxidase reaction. ×100. (F) Same as Fig. 3A. **Localized AL amyloid deposits** are **negative for** antihuman **amyloid A component** 1:100 (monoclonal antibody MO759; DAKO, Glostrup, Denmark), Streptavidin-biotin complex/horseradish peroxidase reaction. ×100.

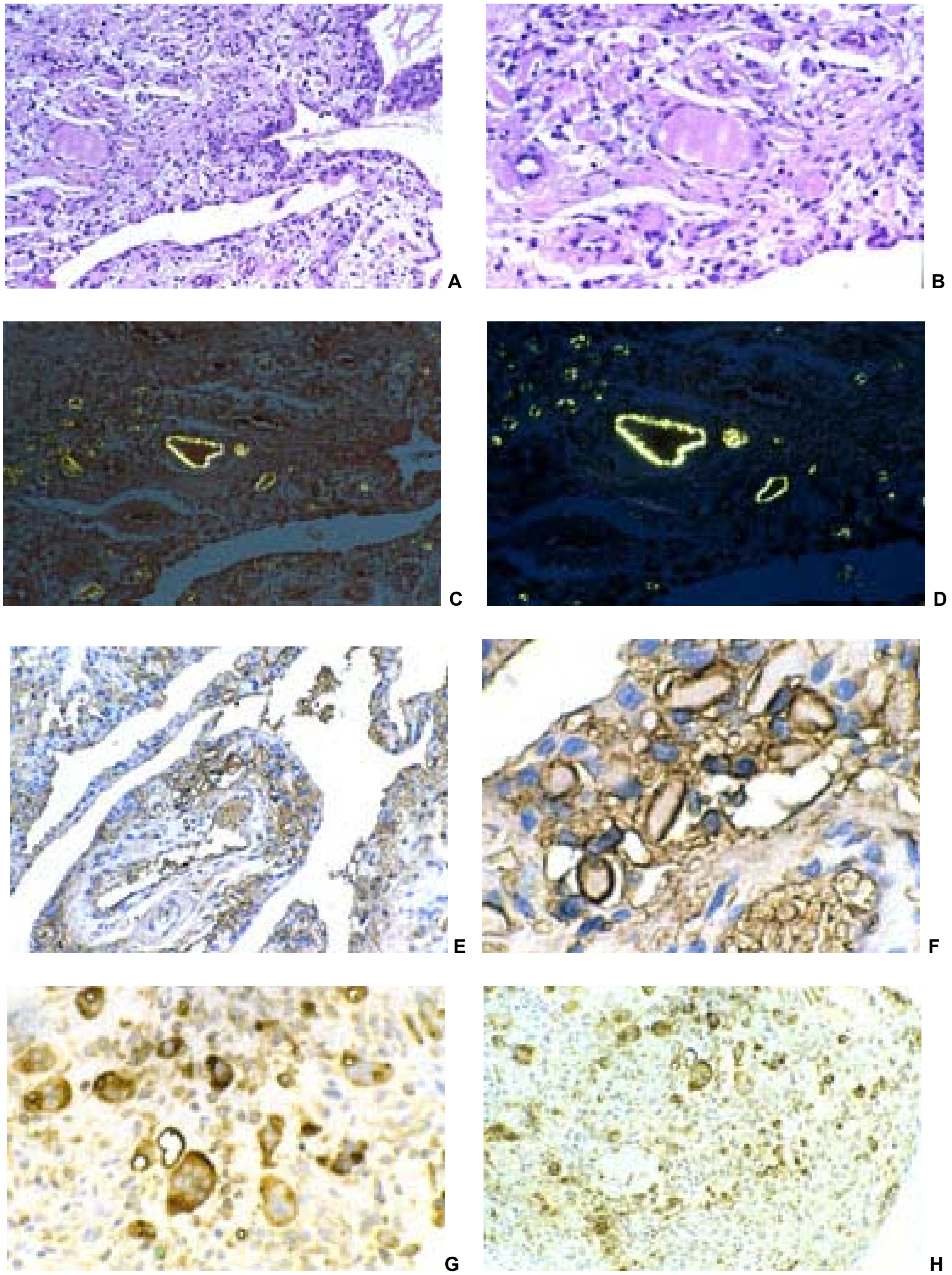

**FIGURE 4A-H.** Synovial membrane. Systemic β2-microglobulin (Aβ2M) amyloidosis. (A,B) Aβ2M deposits, extracellular and incorporated by macrophages. Hematoxylin and Eosin, (A) ×125, (B) ×200. (C,D) Performate pretreatment for 3 sec, Congo red staining, viewed under polarized light. **Aβ2M deposits** are **resistant** to **performate pretreatment** (**for 1-15 sec),** (C) ×125, (D) ×200. (E,F) Systemic **Aβ2M** deposits are **positive** for a-hu β2-microglobulin 1:400 (polyclonal antibody A0072; DAKO, Glostrup, Denmark), Streptavidin-biotin complex/horseradish peroxidase reaction, (E) ×50, (F) ×200. (G,H) Synovial membrane, macrophages with incorporated β2-microglobulin. Positive staining for a-hu β2-mikroglobulin 1:400 (polyclonal antibody A0072; DAKO, Glostrup, Denmark), Streptavidin-biotin complex/horseradish peroxidase reaction, (G) ×125, (H) ×200.

### CONCLUSION

Degradation of amyloid deposits by performate treatment according to Romhányi (1979) or by $KMnO_4$ oxidation according to Wright (1977), followed by Congo red staining according to Romhányi (1971), and viewed with polarized light is a practical and easy method in differentiation of most frequent types of amyloidosis. Different times of degradation according to Bély and Apáthy[8,9] allow a more precise distinction of various amyloid deposits.

Amyloid deposits may be disintegrated by other oxidative agents as well, for example, by peracetate or by alkaline solutions[13].

Immunohistochemical staining of AA amyloid deposits shows a definite positive reaction for the antihuman amyloid A component. It is an excellent and reliable staining procedure.

The immunohistochemical evaluation of AL amyloid deposits may be difficult because of the intensive background staining.

Hereditary forms of amyloidosis should be excluded by medical history, or by biochemical and molecular biologic methods. (Unfortunately we have no personal experience with histochemically differentiating transthyretin amyloidosis).

The organ (tissue)–limited, isolated types of amyloid (***Aβ***, ***AIAPP***, ***Aβ2M***) deposits may be differentiated by classical histology, or based on their typical localization (brain, islets of Langerhans, articular cartilage, tendons, lung, prostate, etc.).

The histochemical analysis (the time-dependent degradation) of amyloid deposits differentiates between early "fresh" and late stage, massive "old" amyloid deposits. Old amyloid deposits need longer time for degradation with a given oxidative agent.

None of the currently used immunohistochemical stains differentiates between early and late stage of amyloidosis.

### ACKNOWLEDGMENT

The Olympus BX51 polarized light microscope was generously provided by OTKA Project T/F 046385 of the Hungarian Academy of Sciences.

We are grateful to Professor Dr. Margaret Tzaphlidou, Principal Editor of the Microscopy Domain for TheScientificWorldJOURNAL. This paper would have never been born without her invitation to write a review of the histochemical analysis of amyloidoses.

## APPENDIX

#### Congo red staining according to Romhányi[7]

(Romhányi, G. [1971] Selective differentiation between amyloid and connective tissue structures based on the collagen specific topo-optical staining reaction with congo red. *Virchows Arch*. **354**, 209–222.)

1. Tissues are fixed in 10% neutral buffered formalin, embedded in paraffin – 5-µm sections are cut.
2. Prolonged deparaffinization (3–5 days) in a thermostat at 56°C (changing xylene daily).
3. Chloroform – methanol I. (1:1) solution for 1 h.
4. Chloroform – methanol II. (1:1) solution for 1 h or overnight.
5. 96% alcohol I-II. 30-30 min.
6. Wash in tap water.
7. Rinse in distilled water.
8. Stain in 1% Congo red (distilled water) solution at 20°C for 1 h.
9. Rinse in distilled water 3×5 sec.
10. Pour off distilled water and wipe away the excess.
11. Cover with gum arabic, dry under a Petri dish (in a dust-free place). (Glycerin gelatin may be substituted for gum arabic for better long-term storage.)
12. Mount with Canada balsam coverslip.

#### Performate pretreatment – Congo-red staining according to Romhányi[5]

(Romhányi, G. [1979] Selektive Darstellung sowie methodologische Möglichkeiten der Analyse ultrastruktureller Unterschiede von Amyloidablagerungen. *Zentralbl. Allg. Pathol. Pathol. Anat.* **123**, 9–16.)

1. Tissues are fixed in 10% neutral buffered formalin embedded in paraffin – 5-µm sections are cut.
2. Prolonged deparaffinization (for 3–5 days) in a thermostat at 56°C.
3. Chloroform – methanol I. (1:1) solution for 1 h.
4. Chloroform – methanol II. (1:1) solution for 1 h or overnight.
5. Wash in 96% alcohol I-II. 30-30 min.
6. Wash in tap water, rinse in distilled water.
7. Amyloid is demonstrated and characterized histochemically by Congo red staining after performate pretreatment.
8. 85% HCOOH; 8 ml.
9. 30% $H_2O_2$; 3 ml.
10. 96% $H_2SO_4$; 0.22 ml.
11. Pretreatment at 20°C for 1, 5, 10, 20 sec.
12. Rinse in distilled water 2×5 min.
13. Stain in 1% Congo red (distilled water) solution at 20°C for 1 h.
14. Rinse in distilled water 3×5 sec.
15. Pour off distilled water and wiper away the excess.
16. Cover with gum arabic, dry under a Petri dish (in a dust-free place).
17. Mount with Canada balsam coverslip.

#### Permanganate method according to Wright[6]

(Wright, J.R., Calkins, E., and Humphrey, R.L. [1977] Potassium permanganate reaction in amyloidosis. *Lab. Invest.* **36**, 274–281.)
(In *Histochemistry Theoretical and Applied. Vol. 2. Analytical Technology*. Pearse, A.G.E., Ed. Churchill Livingston, Edinburgh. 1985, p. 598.)

1. Tissues are fixed in 10% neutral buffered formalin embedded in paraffin – 5-µm sections are cut.
2. Prolonged deparaffinization (3–5 days) in a thermostat at 56°C (changing xylene daily).
3. Chloroform – methanol I. (1:1) solution for 1 h.
4. Chloroform – methanol II. (1:1) solution for 1 h or overnight.

5. 96% alcohol I-II. 30-30 min.
6. Wash in tap water.
7. Rinse in distilled water.
8. Immerse sections in 5% aqueous $KMnO_4$ with 0.3% $H_2SO_4$,
9. For 30 sec, and 1, 3, 6, 10, 15, 20 min.
10. Decolorize with 5% oxalic acid.
11. Wash twice in distilled water.
12. Stain in 1% Congo red (distilled water) solution at 20°C for 1 h.
13. Rinse in distilled water 3×30 sec.
14. Tap off distilled water and wipe away the excess.
15. Cover with gum arabic, dry under a Petri dish (in a dust-free place).
16. Mount with Canada balsam coverslip.
